# Investigation into Incidence of Albuminuria and Casts in British Soldiers during Training and the Relationship of This Condition to War Nephritis

**Published:** 1919-01

**Authors:** 


***Investigation Into Incidence of Albuminuria and Casts in British Soldiers During Training and the Relationship of this Condition to War Nephritis.*** Capt. H. MAC LEAN, R. A. M. C. *Journal of the Royal Army Medical Corps*, September 1918.

A series of investigations regarding the minimum incidence of albuminuria and casts in soldiers undergoing training was made at Aldershot and at a British Base in France. In all 60 000 men were examined and the results recorded as follows :

*General Procedure in Collection and Examination of Urine.* Morning urine alone was examined and 2 groups of 300 men were done each day. On the previous evening, the men had to fill in special forms, giving their name, rank, age, etc., and stating whether they had ever had scarlet fever, gonorrhea, syphilis or any other illness. Urine was collected in special numbered tins, and all positive specimens (about 50 o/o) were sent to the laboratory, graded according to the amount of protein present, centrifuged and examined for casts.

*Tests Used for Protein and Methods of Grading Urines According to Amount of Albumin.* The testing reagent was salicyl-sulphonic acid, and, in cases of doubt, a further examination was made by heat coagulation and cold nitric acid.

*Albuminuria and War Nephritis.* It has been impossible, so far, to establish with certainty the existence of a relationship between war nephritis and albuminuria : several men who, on examination at the base, gave no indication of albuminuria, have subsequently returned with acute nephritis; on the other hand, the same occurred in cases of men already suffering from albuminuria. The results of the present investigation, however, strongly support the view that the albuminuria of soldiers in active training at the base is not increased by the conditions associated with this training.

*Incidence of Albuminuria in 5000 Men Examined at the Base.* The actual figures of 50 000 men in training were as follows :

| | |
|---|---|
| Total number with albuminuria . . . . . . . . | 6.48 o/o |
| Number with albuminuria after allowing for pus, spermatozoa, etc. . . . . . . . . . . . . . . . . | 5.62 o/o |
| Number with gross albuminuria. . . . . . . . | 2.55 o/o |
| Number with gross albuminuria after allowing for pus, etc. . . . . . . . . . . . . . . . . . | 2.29 o/o |

*Incidence of Albuminuria Among Soldiers in the Trenches.* The following observations have been recorded by Mac Leod :

| | |
|---|---|
| *a*) French troops, examined within 24 hours after return from the trenches. . . . . . . . . | 1.66 o/o |
| *b*) British troops the majority of whom had been in the trenches recently . . . . . . . . . | 4.73 o/o |
| *c*) British troops after 12 months in France. . . | 3.53 o/o |

*Presence of Casts in Albuminous Urines.*

*General Procedure.* About 25 cc. were centrifuged for a few minutes, and the deposit examined under the microscope. It was found convenient to pipette approximately 1/2 cc. of material from the bottom of the centrifuge glass, and to spread this in a thin layer over the greater part of an ordinary microscope slide. It was then examined quickly with a low power.

*Nature of Casts Present.* Almost all the casts found were either hyalin or epithelial; very few blood casts were encountered. In the majority of cases, epithelial casts showed the usual appearences seen in acute nephritis; hyalin casts comprised, in addition to a number of the ordinary clear, ill-defined variety, a considerable amount of hyalo-granular casts. With the exception of certain cases of acute nephritis, the epithelial cast is more seldom present than the hyalin.

*General Results Obtained.* For 50 000 men examined, the average percentage of urines found to contain casts was 1.87 o/o; of this number 0.84 o/o had definite epithelial casts, while in 1.03 o/o hyalin casts only were found.

